# Indomethacin testing in suspected paroxysmal hemicrania and hemicrania continua: Clinical phenotypes and response patterns in a tertiary headache centre

**DOI:** 10.1007/s10072-026-09456-9

**Published:** 2026-10-06

**Authors:** Antoine Bender, Mirela Muresan, Lauriane Joganah, Eric Salvat

**Affiliations:** 1 Centre d’Evaluation Et de Traitement de La Douleur, Hôpitaux Universitaires de Strasbourg, Strasbourg, France; 2 Service de Neurologie, Hôpitaux Universitaires de Strasbourg, Strasbourg, France; 3 Cognitive and Adaptive Neuroscience Laboratory, Centre National de La Recherche Scientifique, University of Strasbourg, Strasbourg, France

**Keywords:** Case series, Hemicrania continua, Paroxysmal hemicrania, Headache, Indomethacin, Indomethacin trial, Secondary headache, ICHD-3

## Abstract

**Background:**

Indomethacin responsiveness is considered the defining diagnostic feature of hemicrania continua and paroxysmal hemicrania. However, the clinical presentation of indomethacin-responsive headaches is often heterogeneous, and practical guidance regarding which patients should undergo an indomethacin trial, the optimal duration of treatment, and the interpretation of partial responses remains limited.

**Cases:**

Eighteen patients underwent an indomethacin trial in our pain centre over the last year, of whom eleven (61%) achieved a positive response. Positive responders included patients ultimately diagnosed with probable or definite hemicrania continua (*n* = 6); and probable or definite paroxysmal hemicrania (*n* = 5), including secondary forms. Clinical presentations frequently departed from ICHD-3 criteria. Trigeminal autonomic symptoms were absent in several responders, while migrainous features were common. Attack duration in patients with paroxysmal hemicrania frequently exceeded the 30-min upper limit defined by ICHD-3, and some patients presented with pain characteristics suggestive of neuropathic disorders. The median time to a clinically meaningful response was 13 days for hemicrania continua (range 3–35 days) and 11 days for paroxysmal hemicrania (range 4–23 days). Most patients responded to 150 mg/day, although escalation to 225 mg/day or prolonged treatment exceeding four weeks was occasionally required. Seven patients had negative trials, leading to alternative diagnoses including migraine, cluster headache, SUNA, post-traumatic headache, and idiopathic mandibular neuropathy.

**Conclusions:**

This case series illustrates the broad clinical spectrum of indomethacin-responsive headaches and highlights the limitations of relying exclusively on ICHD-3 criteria when selecting patients for an indomethacin trial. Clinical suspicion should remain broad, particularly in patients with strictly unilateral headaches, atypical trigeminal autonomic cephalalgias, or refractory unilateral primary headaches.

**Supplementary Information:**

The online version contains supplementary material available at https://doi.org/10.1007/s10072-026-09456-9.

## Introduction

Trigeminal autonomic cephalalgias (TACs), with cluster headache as their prototypical disorder, comprise a heterogeneous group of primary headache disorders. They can be broadly categorized into indomethacin-responsive and indomethacin-unresponsive entities. Paroxysmal hemicrania and hemicrania continua are considered the hallmark indomethacin-responsive headaches, both being defined by an absolute response to therapeutic doses of indomethacin [1].

Hemicrania continua (HC) is characterized by a continuous unilateral headache, which is side-locked in most patients, with superimposed exacerbations accompanied by cranial autonomic symptoms. The duration and frequency of exacerbations vary considerably, both across patients and within the same individual over time. A recent systematic review highlighted the marked clinical heterogeneity of the disorder, reporting exacerbations lasting from a few seconds to several weeks. Cranial autonomic symptoms are absent in approximately one-quarter of patients, whereas more than half fulfil the International Classification of Headache Disorders, 3rd edition (ICHD-3), diagnostic criteria for migraine during exacerbations [2].

Paroxysmal hemicrania (PH) is characterized by recurrent attacks of severe unilateral pain without an underlying continuous headache. Although attacks are strictly unilateral, the side may alternate over the course of the disease. Cranial autonomic symptoms accompany most attacks. According to the ICHD-3, attacks last between 2 and 30 min, partially overlapping in duration with cluster headache attacks (15–180 min). Cranial autonomic symptoms are absent in only around 10% of patients but may occur independently of pain in up to one-third of cases. In addition, approximately one-quarter of patients exhibit migrainous features during attacks [3].

An absolute response to indomethacin is considered the defining feature of both disorders. Indomethacin is a non-steroidal anti-inflammatory drug (NSAID) with preferential cyclooxygenase-1 (COX-1) inhibition. However, other NSAIDs, including selective COX-1 inhibitors, fail to reproduce the complete and sustained therapeutic efficacy observed with indomethacin. Several alternative mechanisms have therefore been proposed, including modulation of cerebral blood flow and reduction of intracranial pressure. Nevertheless, the biological mechanisms underlying the unique efficacy of indomethacin in these disorders remain unknown [4].

Despite the central role of indomethacin in the diagnosis of paroxysmal hemicrania and hemicrania continua, several practical aspects of its use remain poorly defined. The optimal duration of an indomethacin trial before concluding treatment failure, the dose required to establish a negative response, and the clinical phenotypes warranting an indomethacin trial have not been clearly established.

Here, we present a consecutive case series reflecting the experience of a specialized headache consultation within a tertiary pain assessment and management centre. We report 18 patients who underwent an indomethacin trial over the last year, with the aim of further characterizing the clinical spectrum of these disabling yet frequently under-recognized disorders and informing the practical use of indomethacin in routine clinical practice. By describing the clinical presentation, treatment response and temporal profile of indomethacin responsiveness, we aim to refine the practical approach to indomethacin testing and contribute to a better recognition of these uncommon but likely underdiagnosed headache disorders.

## Cases

Patients with a clinical presentation suggestive of paroxysmal hemicrania (PH) or hemicrania continua (HC) were considered to have definite PH/HC if they exhibited an absolute response to indomethacin. In patients with a clinical phenotype suggestive of PH/HC but without complete response, the indomethacin test was considered positive when a clinically meaningful improvement was observed, defined using a 70% threshold reduction in headache burden. Patients achieving a ≥ 70% but < 100% response were classified as having probable PH/HC, reflecting a high degree of diagnostic suspicion despite an incomplete response to indomethacin. This threshold was chosen to minimize the inclusion of nonspecific improvements attributable solely to the anti-inflammatory effects of the drug which may also be observed with other medications, and to avoid dismissing the diagnosis in patients with a suggestive phenotype who respond only to indomethacin.

All patients underwent brain MRI either before referral or following a prescription at the initial consultation. Whenever imaging abnormalities were identified, they are described together with the corresponding patient cases. Unless otherwise specified, brain MRI findings were unremarkable.

In six patients, the indomethacin trial was initiated because of a clinical presentation compatible with hemicrania continua according to the ICHD-3 criteria [1], notably strictly unilateral headache associated with trigeminal autonomic symptoms or restlessness. Four patients had a positive response, whereas two had a negative response.

In six patients, the indomethacin trial was initiated because of a clinical presentation compatible with paroxysmal hemicrania according to the ICHD-3 criteria [1], notably strictly unilateral headache associated with trigeminal autonomic symptoms or restlessness. Three patients had a positive response and three had a negative response.

In four patients, the indomethacin trial was initiated because of strictly unilateral headache despite the absence of trigeminal autonomic symptoms or restlessness. Two patients had a positive response and two had a negative response.

Finally, in two patients, the indomethacin trial was performed because of an unusual resistance to acute and preventive treatments for the initially suspected headache disorder. One of these patients presented with side-shifting unilateral headaches. Both patients had a positive response to indomethacin.

The presence or absence of associated migrainous features, particularly during exacerbations, did not influence the decision to initiate an indomethacin trial, given the frequency of migrainous features in paroxysmal hemicrania and hemicrania continua [3].

### Patients with negative indomethacin trials

A detailed description of each case is provided in the Appendix.

Seven of the 18 patients underwent an indomethacin trial that was ultimately considered negative (Table 1). Final diagnoses included chronic migraine (*n* = 3), persistent headache attributed to mild traumatic injury to the head (*n* = 1), episodic cluster headache (*n* = 1), secondary SUNA associated with multiple sclerosis (*n* = 1), and probable idiopathic painful mandibular neuropathy (*n* = 1).

**Table 1:** Clinical characteristics of patient with a negative indomethacin trial

Patient		1	2	3	4	5	6	7
Sex		F	M	F	F	M	F	M
Age at symptom onset		27	45	36	29	29	31	50
Side affected		Left	Right	Left	Left	Right	Right	Left
Conti-nuous pain	Intensity	/	4/10	2/10	/	/	2/10	4/10
	Type	/	Pressure	Pressure	/	/	Discomfort	Pressure
	Localisation	/	Temporal and periorbital	Parietal	/	/	Ophthalmic and maxillary trigeminal divisions	Mandibular division of trigeminal nerve
Exacer-bation	Intensity	7/10	8/10	6/10	8/10	10/10	10/10	8/10
	Duration	2 h	1 h	5 h	12 h	30 min	< 3 min	Several hours
	Type	Pressure	Crushing	Pulsatile	Pulsatile	Crushing	Stabbing, electric shock-like	Burning and tearing
	CAS	No	Conjunctival injection	No	Ipsilateral lacrima-tion and rhinorrhea	Eyelid oedema	Ipsilateral lacrimation, ptosis	No
	Restless-ness	No	No	No	No	Yes	Yes	Yes
	Migrainous feature	Nausea	No	Photo-phono-phobia	Photo-phono-phobia and nausea	No	No	No
Dose of indomethacin (in mg per day)		225mg	75mg	150mg	150mg	300mg	225mg	225mg
Duration of indomethacin		2 weeks	2 days	4 weeks	4 weeks	4 weeks	3 weeks	4 weeks
Final diagnosis (code ICHD-3)		Probable migraine (1.5)	Persistent headache attributed to mild traumatic injury to the head (5.2.2)	Chronic migraine with central sensi-tization (1.3)	Chronic migraine with cranial autonomic symptoms (1.3)	Episodic cluster headache (3.1.1)	SUNA (3.3.2)	Probable painful idiopathic trigeminal neuro-pathy (13.1.2.5)

*F* Female; *M* Male;/: absent; *CAS* Cranial autonomic symptoms; *ICHD-3* International classification of headache disorders 3rd edition

One patient exhibited a partial response to indomethacin, with an approximately 30% reduction in attack frequency and intensity after two weeks at 150 mg/day, without additional benefit following dose escalation to 225 mg/day. An identical improvement was subsequently observed during a scheduled trial of naproxen sodium, suggesting a non-specific NSAID effect rather than true indomethacin responsiveness. The final diagnosis was revised to probable migraine, and the patient subsequently achieved substantial improvement with onabotulinumtoxinA according to the PREEMPT protocol.

In one patient with a post-traumatic unilateral continuous headache clinically resembling hemicrania continua, assessment of indomethacin responsiveness was precluded by poor tolerability. Disabling nausea and dizziness occurred despite treatment with only 25 mg three times daily combined with proton pump inhibitor prophylaxis, preventing adequate dose escalation. The final diagnosis was persistent headache attributed to mild traumatic injury to the head (ICHD-3 code 5.2.2) while not excluding probable hemicrania continua due to poor tolerability of indomethacin.

Two patients with refractory chronic migraine underwent an indomethacin trial because of atypical unilateral presentations. One had a continuous unilateral background headache suggestive of hemicrania continua, whereas the other presented with prominent ipsilateral lacrimation and rhinorrhea during attacks, raising suspicion of paroxysmal hemicrania. Neither patient experienced any benefit after one month of treatment at 150 mg/day, and both reported greater efficacy with conventional NSAIDs than with indomethacin. Both were ultimately diagnosed with chronic migraine.

One patient with a previous diagnosis of episodic cluster headache underwent an indomethacin trial because of the relatively short attack duration, raising the possibility of paroxysmal hemicrania. Despite dose escalation to 300 mg/day over one month, attack frequency decreased by only 20%, and the trial was considered negative.

Another patient with relapsing–remitting multiple sclerosis presented with recurrent short-lasting unilateral attacks accompanied by cranial autonomic symptoms, clinically consistent with SUNA. Indomethacin administered at 150 mg/day for one week followed by 225 mg/day for three weeks produced no clinical benefit. The final diagnosis was secondary SUNA associated with multiple sclerosis.

The final patient presented with continuous unilateral pain confined to the left mandibular division of the trigeminal nerve, associated with prolonged exacerbations with neuropathic characteristics and restlessness but without cranial autonomic symptoms. Because of the strictly unilateral phenotype and the failure of multiple neuropathic pain treatment, an indomethacin trial was performed up to 225 mg/day for four weeks without benefit. The final diagnosis was probable idiopathic painful mandibular neuropathy.

### Patients with positive indomethacin trials

A detailed description of each case is provided in the Appendix.

Eleven of the 18 patients achieved a positive response to indomethacin according to our criterion (> 70% clinical improvement), and eight of the eleven patients achieved an absolute response (Tables 2 and 3). Final diagnoses included hemicrania continua (*n* = 6), paroxysmal hemicrania (*n* = 5), including one probable secondary to moderate traumatic brain injury, and one probable secondary hemicrania continua associated with treated intracranial metastatic disease.

**Table 2:** Clinical characteristics of patient with a positive indomethacin trial (patients 1 to 6)

Patient		1	2	3	4	5	6
Sex		F	F	M	M	F	F
Age at symptom onset		40	79	26	33	43	32
Side affected		Right	Right	Left	Both	Right	Right
Conti-nuous pain	Intensity	3/10	5/10	/	/	4/10	/
	Type	Pressure	Pressure	/	/	Dragging sensation	/
	Localisation	Temporo-parietal	Hemicranium	/	/	Hemicranium	/
Exacer-bation	Intensity	9/10	10/10	10/10	10/10	10/10	7/10
	Duration	1 to 4 days	Few hours	30 min	45 min	Several hours to several days	30 min to 8 h
	Type	Crushing and electric shock-like	Tearing	Crushing	Crushing	Crushing and vertigo	Pulsatile
	CAS	No	No	Lacrimation, ptosis, conjuncttival injection	No	Ptosis, conjuncttival injection	No
	Restless-ness	No	No	No	Yes	No	Yes
	Migrainous feature	Photo-phono-phobia	Photo-phobia	No	No	No	Photo-phono-phobia, nausea
Dose of indomethacin (in mg per day)		150mg	150mg	150mg	225mg	150mg	225mg
Time to response		8 days	10 days	4 days	11 days	16 days	21 days
Initial suspected diagnosis		Chronic migraine	Headache attributed to intracranial neoplasia	Chronic cluster headache	Chronic cluster headache	Hemicrania continua	Persistent post-traumatic headache
Final diagnosis (code ICHD-3)		Hemicrania continua (3.4.2)	Secondary hemicrania continua (3.4)	Paroxysmal hemicrania (3.2.2)	Paroxysmal hemicrania (3.2.2)	Hemicrania continua (3.4)	Secondary paroxysmal hemicrania (3.2)

*F* Female; *M* Male;/: absent; *CAS* Cranial autonomic symptoms; *ICHD-3* International classification of headache disorders 3rd edition

**Table 3:** Clinical characteristics of patient with a positive indomethacin trial (patients 7 to 11)

Patient		7	8	9	10	11
Sex		F	F	M	F	M
Age at symptom onset		31	50	38	33	42
Side affected		Right	Left	Left	Left	Right
Conti-nuous pain	Intensity	4/10	6/10	3/10	/	/
	Type	Burning, tingling, painful cold	Burning, tingling, electric shock-like	Pulsatile, circadian	/	/
	Localisation	Temporo-parietal	Cheek and retro-auricular	Temporo-parietal	/	/(occipital)
Exacer-bation	Intensity	8/10	10/10	8/10	10/10	6/10
	Duration	2 weeks	< 5 min	Few minutes to several days	Several days	1 to 2 h
	Type	Burning, tingling, electric shock-like	Burning, tingling, electric shock-like	Crushing	Pulsatile, pulling	Pulsatile, pressing
	CAS	Ipsilateral facial erythema	No	Lacrimation, ptosis, hemi-facial sweating	No	No
	Restless-ness	No	Yes	No	No	Yes
	Migrainous feature	No	No	Photo-phono-phobia	Photo-phono-phobia, nausea, vomiting	Photophobia
Dose of indomethacin (in mg per day)		150mg	150mg	375mg	225mg	150mg
Time to response		3 days	4 weeks	5 weeks	23 days	9 days
Initial suspected diagnosis		Neuropathic pain	Neuropathic pain	Hemicrania continua	Chronic migraine	Occipital neuralgia
Final diagnosis (code ICHD-3)		Hemicrania continua (3.4.2)	Hemicrania continua (3.4.2)	Hemicrania continua (3.4)	Paroxysmal hemicrania (3.2.2)	Paroxysmal hemicrania (3.2)

*F* Female; *M* Male;/: absent; *CAS* Cranial autonomic symptoms; *ICHD-3* International classification of headache disorders 3rd edition

Among the six patients diagnosed with probable and definite hemicrania continua, four presented with the typical phenotype of a continuous unilateral headache with superimposed exacerbations, whereas one patient had previously been diagnosed with chronic migraine. Cranial autonomic symptoms were absent or minimal in most cases, while migrainous features during exacerbations were frequent. One patient exhibited ipsilateral ptosis and conjunctival injection outside of an exacerbation despite denying autonomic symptoms. Another patient experienced complete resolution of recurrent ictal vertigo following initiation of indomethacin.

Five patients were ultimately diagnosed with probable or definite paroxysmal hemicrania. Two had previously been diagnosed with treatment-refractory cluster headache after failure of high-dose verapamil, lithium, triptans and, in one case, oxygen therapy. One patient developed probable paroxysmal hemicrania following moderate traumatic brain injury. Two additional patients had initially been referred with presumed idiopathic neuropathic pain because of continuous unilateral facial pain and neuropathic pain characteristics. Indomethacin completely resolved symptoms in both cases, leading to diagnostic revision.

Time to response was highly variable. Complete or near-complete improvement occurred within the first week in some patients, whereas others required more prolonged treatment before responding. Clinical benefit was observed after 8, 16, 21 and 23 days in four patients, and one patient required escalation to 375 mg/day for five weeks before complete symptom resolution. Conversely, two patients achieved complete remission within four days of treatment initiation.

Long-term follow-up also demonstrated heterogeneous treatment courses. Several patients remained asymptomatic after dose reduction to maintenance doses ranging from 75 to 150 mg/day. However, tapering attempts resulted in prompt symptom recurrence in several individuals, within 24 to 48 h, which has been considered by some authors to be more specific for this type of headache than the response to an indomethacin therapeutic trial [2]. One patient discontinued indomethacin because of suspected gastrointestinal toxicity despite complete clinical remission, with headache recurrence occurring within 48 h. Another patient developed secondary loss of efficacy after five months despite escalation to 375 mg/day, ultimately requiring alternative preventive therapies. Finally, one elderly patient receiving anticoagulant therapy experienced complete headache remission at a relatively low dose, but treatment was discontinued because of suspected gastrointestinal adverse events.

**Fig. 1:**
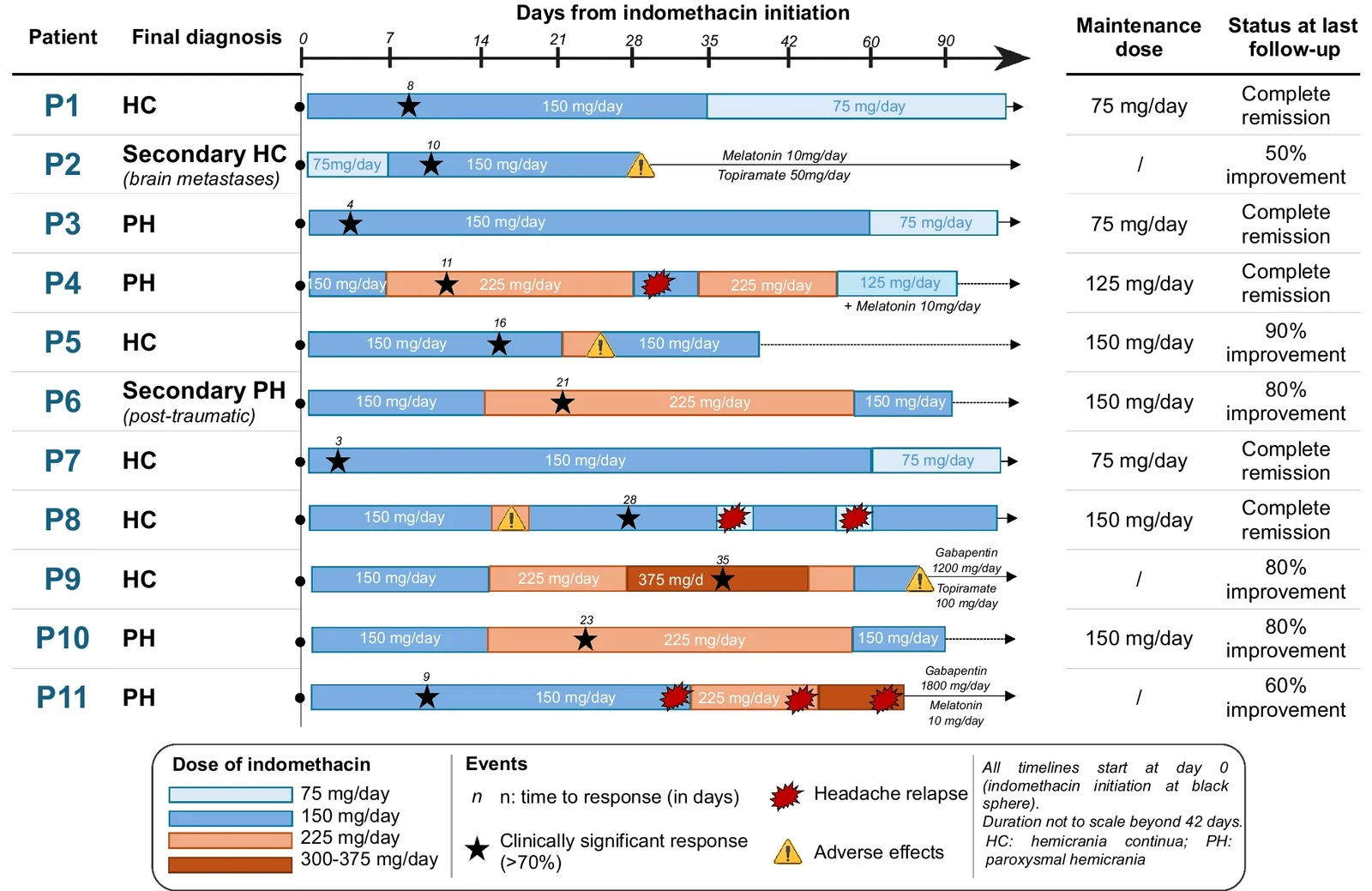
Clinical timelines of the 11 patients with a positive indomethacin trial. Maintenance doses represent the lowest dose that maintained satisfactory control at last follow-up. Dotted arrow was used when patient did not reach a 90-day follow-up yet

Median time to response was 11 days (range 3–35). Five patients responded after more than two weeks, and four required escalation to 225 mg/day or more before achieving complete remission. A clinical timeline of the 11 patients with a positive indomethacin trial is provided in the Fig. 1.

Among patients with probable or definite hemicrania continua, 83% were female, with a median age of 41.5 years. Two-thirds presented with strictly right-sided pain, whereas one-third had strictly left-sided pain. The median baseline pain intensity was 3.5/10, while the median peak intensity during exacerbations was 9.5/10. The duration of exacerbations showed marked inter- and intra-individual variability, ranging from a few minutes to several weeks. Likewise, exacerbation frequency varied considerably, from multiple attacks per day to a single exacerbation every few months. Cranial autonomic symptoms were present in 50% of patients, whereas agitation was reported in 16%. One-third of patients exhibited neither cranial autonomic symptoms nor agitation. Migrainous features during exacerbations were present in 50% of cases. All but one patient achieved a clinically meaningful response with indomethacin at a dosage of 150 mg/day. The median time to response was 13 days (range, 3–35 days).

**Fig. 2:**
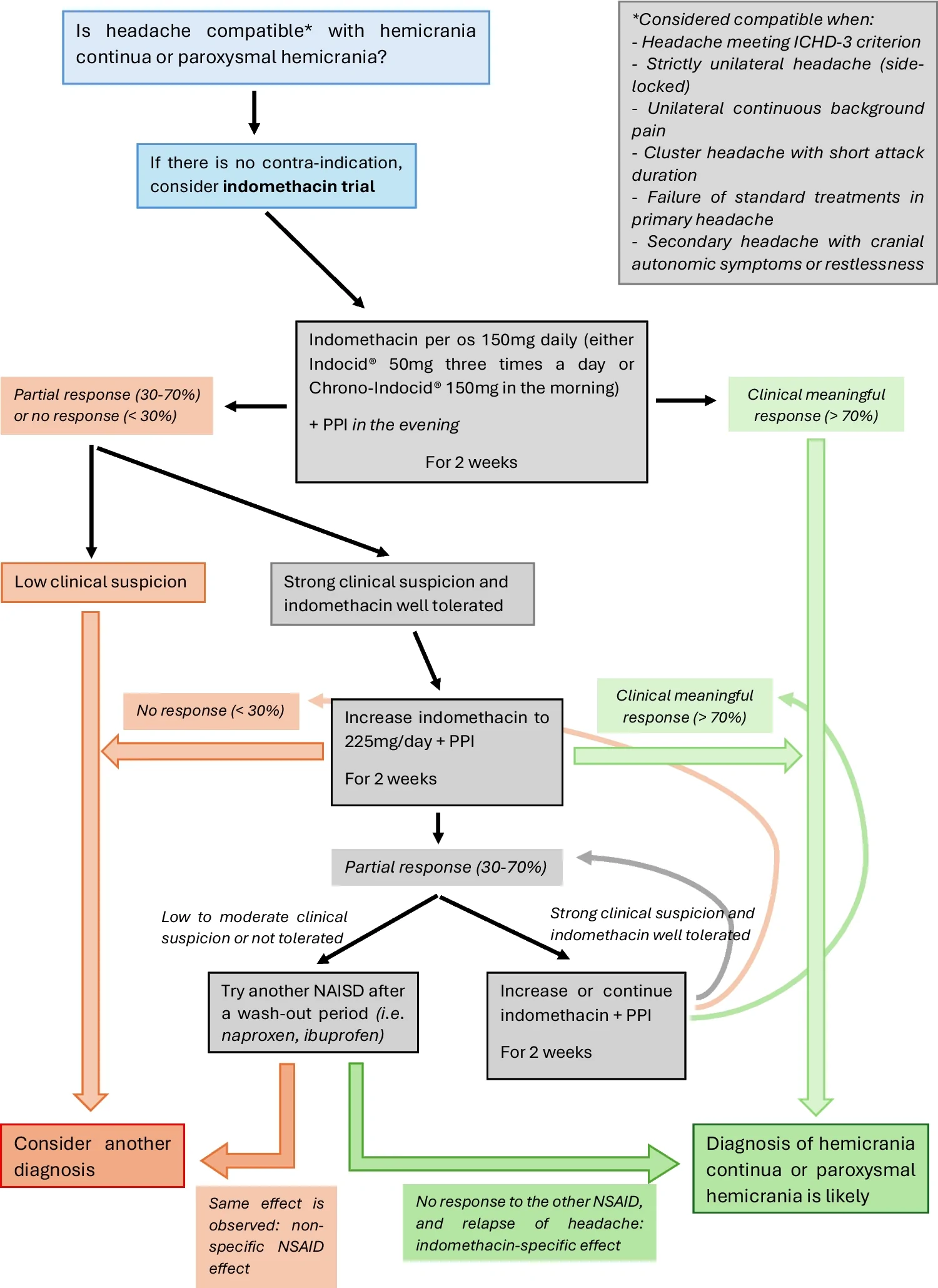
Illustrative representation of the institutional approach to indomethacin treatment at our center. This figure is descriptive only and does not represent an evidence-based treatment recommendation

Among patients with probable or definite paroxysmal hemicrania, 60% were male, with a median age of 33 years. Eighty percent experienced strictly unilateral attacks, equally divided between the left and right sides, whereas one patient reported side-alternating attacks. The median pain intensity during attacks was 10/10. Excluding the patient whose exacerbations lasted several days, the median attack duration was 67.5 min. Forty percent of patients experienced daily or multiple daily attacks, whereas the remaining 60% had fewer than one attack per day but still experienced several attacks per week. Cranial autonomic symptoms were documented in only one patient; however, this may represent underreporting, as the extreme pain intensity may have limited patients' awareness of subtle accompanying autonomic features. Agitation during attacks was reported in more than half of the patients, and migrainous features were also present in more than half. More than half of the patients achieved a clinically meaningful response with indomethacin at a dose of 150 mg/day, whereas two patients required dose escalation to 225 mg/day. The median time to response was 11 days (range, 4–23 days).

Given the limited sample size, these descriptive findings should be considered hypothesis-generating rather than representative of the broader population.

## Discussion

### Main findings

In this retrospective case series, 11 of 18 patients experienced a clinically meaningful response to indomethacin and eight of eleven patients exhibited an absolute response to indomethacin. As expected, responders were ultimately diagnosed with hemicrania continua or paroxysmal hemicrania, including two probable secondary cases. However, our observations extend beyond confirming the diagnostic utility of indomethacin. They illustrate the marked phenotypic heterogeneity of indomethacin-responsive headaches, the limitations of current diagnostic criteria in identifying candidates for treatment, and the practical challenges involved in defining an adequate therapeutic trial.

As described in other series, several responders lacked cranial autonomic symptoms, while others fulfilled migrainous criteria during exacerbations [3]. Two patients had previously been diagnosed with treatment-refractory cluster headache, one with chronic migraine, and two with presumed idiopathic neuropathic facial pain. Conversely, several patients with equally convincing unilateral phenotypes did not respond to indomethacin and were ultimately diagnosed with chronic migraine, post-traumatic headache, SUNA, cluster headache or painful trigeminal neuropathy. Given the suggestive clinical phenotype, we cannot exclude the possibility that some patients may have had indomethacin-insensitive hemicrania, as previously suggested by some authors, or that diagnostic certainty may have been limited by poor tolerability of indomethacin [5]. These findings suggest that atypical clinical presentations should not discourage an appropriately conducted therapeutic trial of indomethacin: it emphasizes the need for an indomethacin trial before a side-locked headache, or before patients with a unilateral primary headache disorder refractory to standard treatments [6].

### Limitation of current diagnosis criterion

The ICHD-3 criteria depict a relatively characteristic clinical phenotype, with strictly unilateral attacks, either short-lasting or superimposed on a continuous background headache, accompanied by cranial autonomic symptoms and/or restlessness. In routine clinical practice, however, patients rarely fulfil every diagnostic criterion. Moreover, even patients with highly typical presentations may initially have a negative indomethacin trial because of poor drug tolerance, insufficient treatment duration, or inadequate dosing. In our series, several patients with ultimately positive indomethacin trials exhibited neither cranial autonomic symptoms nor restlessness, whereas others fulfilled the ICHD-3 diagnostic criteria for migraine during exacerbations.

Among patients diagnosed with paroxysmal hemicrania, attack duration frequently exceeded the current ICHD-3 upper limit of 30 min. Furthermore, pain quality may be misleading, with some patients describing burning, stabbing, or electric shock-like pain suggestive of a neuropathic disorder. Similar case of hemicrania mimicking trigeminal neuralgia have previously been reported [7]. Considerable clinical overlap was also observed between cluster headache and paroxysmal hemicrania. In our opinion, patients with a clinical diagnosis of cluster headache who fail to respond to standard acute and preventive therapies should routinely undergo an indomethacin trial. Likewise, an indomethacin trial should be considered in patients presenting with an atypical unilateral headache syndrome or with a unilateral primary headache refractory to standard evidence-based treatments.

Finally, an increasing number of secondary hemicrania cases—including post-traumatic forms—have been described in the literature [8, 9]. Although current evidence suggests that secondary hemicranias respond to indomethacin similarly to primary forms, available data remain limited and larger studies are required.

### Underlying causes found in hemicrania continua and paroxysmal hemicrania

The pathophysiology of hemicrania continua and paroxysmal hemicrania in the events of an underlying cause remains poorly understood. However, functional imaging studies in primary hemicrania continua have demonstrated activation of the posterior hypothalamus, dorsal rostral pons, and other brainstem structures, suggesting involvement of trigemino-autonomic and central pain-processing networks [10]. Secondary hemicrania continua has been reported in association with various structural and systemic conditions, including lung cancer and recent head trauma [11, 12]. The proposed pathophysiology results from activation or dysfunction of trigeminovascular and trigemino-autonomic pathways following traumatic injury or cerebral lesion. However, these mechanisms remain speculative, and the available evidence is largely based on case reports and retrospective observations [13]. The mechanism underlying the marked response to indomethacin in hemicrania continua and paroxysmal hemicrania remains incompletely understood. Beyond cyclooxygenase inhibition, modulation of nitric oxide signaling and trigeminovascular pathways has been proposed, which could explain the specific sensitivity of these phenotypes to indomethacin, including in secondary cases [4, 14].

In our series, one patient had cerebral metastases from lung cancer and another developed hemicrania continua following head trauma, with a temporal relationship in both patients. In the former, the correlation between cerebral metabolic activity on PET and headache severity further suggests a possible link between the underlying lesion and activation of pain-processing networks in our patient, although causality cannot be established.

These observations support the hypothesis that secondary lesions may trigger a hemicrania continua or paroxysmal hemicrania phenotype through modulation of neural pathways involved in primary trigeminal autonomic cephalalgias.

### When to perform an indomethacin trial?

Determining when an indomethacin trial should be performed is arguably the most clinically relevant—and most challenging—question in everyday practice. Although the ICHD-3 diagnostic criteria provide a valuable framework, they should not be interpreted as absolute rules. By introducing the category of probable diagnoses, the classification acknowledges that clinical judgment remains essential when evaluating patients with atypical headache phenotypes.

In our practice, an indomethacin trial is considered whenever a hemicrania is clinically plausible. This includes patients with a strictly unilateral headache, even in the absence of cranial autonomic symptoms or restlessness, as well as patients with side-alternating headaches associated with cranial autonomic features. We also advocate an indomethacin trial in patients presenting with unilateral primary headaches that remain refractory to standard evidence-based therapies.

The threshold for performing an indomethacin trial is even lower in patients with a continuous unilateral background headache accompanied by superimposed exacerbations, regardless of the duration or frequency of these exacerbations, as this phenotype strongly suggests hemicrania continua. In such cases, our experience supports maintaining treatment for longer periods and considering higher doses before concluding that the trial is negative. Likewise, patients presenting with short-duration attacks suggestive of cluster headache should routinely be considered for an indomethacin trial to exclude paroxysmal hemicrania.

Ultimately, the decision to perform an indomethacin trial should be driven by clinical suspicion rather than by strict adherence to ICHD-3 criteria. In the absence of contraindications, whenever the possibility of an indomethacin-responsive headache disorder is seriously entertained, a properly conducted indomethacin trial is likely to provide valuable diagnostic information.

### Dose and duration of indomethacin trial

Neither the optimal duration of treatment nor the maximal dose required before considering the test negative has been clearly established. Existing recommendations are largely based on expert opinion, and published series provide relatively limited information regarding delayed responders.

Our observations suggest that clinically meaningful responses may occur substantially later than commonly anticipated. While some patients achieved complete remission within a few days, several required more than two weeks of treatment before improvement became evident, and one patient only experienced complete symptom resolution after five weeks of treatment at 375 mg/day. Had treatment been discontinued after one or two weeks, this patient would almost certainly have been classified as indomethacin-negative. These findings should be interpreted with caution, given the potential selection bias inherent to recruitment from a tertiary referral centre, where patients are more likely to have treatment-refractory disease. Previous case series have shown that up to 20% of patients may require more than 4 weeks of indomethacin therapy before a clinically meaningful response is observed [15]. Table 4 presents the time to response in our case series.

**Table 4:** Days to clinical meaningful response to indomethacin

Days	Number of patient (%)
Less than 7 days	2 (18)
Between 7 and 14 days	4 (36)
Between 14 and 21 days	3 (27)
Between 21 and 28 days	1 (9)
More than 28 days	1 (9)

Furthermore, some patients achieve substantial symptom improvement rather than complete headache remission, underscoring the marked phenotypic heterogeneity of indomethacin-responsive headache disorders [3, 15].

In clinical practice, the temporal pattern and magnitude of response during an indomethacin trial may be more heterogeneous than implied by the binary diagnostic criterion of “an absolute response to indomethacin”, particularly during dose titration and in patients with atypical phenotypes. Patients with a partial but clinically meaningful response represent a diagnostic grey zone. In these cases, following an appropriate washout period, a comparative trial with another NSAID may help distinguish a specific response to indomethacin from a non-specific anti-inflammatory effect. A similar degree of improvement with another NSAID would argue against an indomethacin-responsive headache disorder, whereas a selective response to indomethacin would strengthen the diagnostic suspicion. The Fig. 2 presents our institutional protocole for illustrative purpose only, as this approach have not been validated in prospective study. 

Although our series is too small to establish evidence-based recommendations, these observations raise the possibility that prematurely interrupted treatment trials or insufficient dose may contribute to underdiagnosis of indomethacin-responsive headaches.

### And after indomethacin?

Our series also illustrates the complexity of long-term management after diagnosis. Although several patients remained symptom-free following dose reduction, others relapsed rapidly after tapering, experienced gastrointestinal intolerance despite proton pump inhibitor prophylaxis, or developed secondary loss of efficacy after several months of treatment. These observations emphasize that confirming the diagnosis represents only the first step, and that individualized long-term treatment strategies remain necessary, particularly in patients unable to tolerate prolonged high-dose indomethacin therapy. Whenever feasible, we used second line treatment to taper or substitute indomethacin [16].

### Limits

This case series has several limitations. Its retrospective single-centre design and limited sample size preclude any estimate of diagnostic performance or treatment efficacy. Referral bias is also likely, as patients were evaluated in a tertiary headache and pain centre where atypical or treatment-refractory cases are overrepresented. In addition, the absence of a standardized indomethacin protocol may have influenced dose escalation and treatment duration. Nevertheless, the series reflects real-world clinical practice and includes a broad range of differential diagnoses encountered when considering an indomethacin trial.

## Conclusion

The question is probably not whether a patient fulfils all ICHD-3 criteria for hemicrania, but whether the clinical presentation is sufficiently suggestive to justify an adequately conducted indomethacin trial. The absence of cranial autonomic symptoms or the presence of migrainous features should not automatically discourage treatment. Likewise, negative conclusions should be drawn cautiously when treatment has been administered for only a short period or at relatively low doses. Prospective multicentre studies are now needed to better define the optimal dose, duration and interpretation of indomethacin trials in suspected hemicrania continua and paroxysmal hemicrania.

## Supplementary Information

Below is the link to the electronic supplementary material.

Supplementary file1 (DOCX 23 KB)

## Data Availability

The data is derived from patient medical records.
