# Supplementary material for: Indomethacin testing in suspected paroxysmal hemicrania and hemicrania continua: Clinical phenotypes and response patterns in a tertiary headache centre
Source: Neurol Sci. 2026 Oct 6;47(10):845. doi: 10.1007/s10072-026-09456-9 (PMC13638709; doi:10.1007/s10072-026-09456-9)
Supplement: Supplementary file 1 [file 10072_2026_9456_MOESM1_ESM.docx]

**Supplementary Appendix – Clinical description of patients with negative indomethacin trials**

**Patient 1**

A 30-year-old woman with no relevant medical history apart from endometriosis had experienced recurrent strictly left-sided headache attacks for the preceding three years. Attacks lasted approximately 2 hours and occurred at a frequency of around three episodes per week. Pain was severe, pressing in quality, non-pulsating, and was not aggravated by physical activity. Nausea was present during most attacks, without vomiting, photophobia, or phonophobia. No cranial autonomic symptoms were reported. All available triptans had previously been ineffective.

Given the clinical presentation, an indomethacin trial was initiated at 150 mg/day together with proton pump inhibitor (PPI) prophylaxis. After two weeks, attack frequency and intensity had improved by approximately 30%. Because of this partial response, the dose was increased to 225 mg/day for an additional two weeks without further benefit. Following a one-month washout period, a scheduled trial of naproxen sodium (550 mg/day for two weeks) resulted in an identical degree of improvement. The indomethacin trial was therefore considered negative; the observed benefit being attributed to a non-specific NSAID effect rather than true indomethacin responsiveness. The diagnosis was revised to probable migraine (attack duration criterion not fulfilled). The patient subsequently achieved substantial clinical improvement with onabotulinumtoxinA administered according to the PREEMPT protocol.

**Patient 2**

A 46-year-old man with well-controlled arterial hypertension and generalized anxiety disorder developed continuous right-sided hemicranial headache with superimposed exacerbations beginning three days after a mild traumatic brain injury. The continuous background pain, rated 4/10 in intensity, involved the right temporal and periorbital regions corresponding to the site of impact. Exacerbations duration were 1 hour, reached an intensity of 8/10 and were accompanied by ipsilateral conjunctival injection. No migrainous features were present.

Cluster headache had initially been suspected by the referring neurologist. Subcutaneous sumatriptan and high-flow oxygen failed to relieve the exacerbations. Verapamil was discontinued because of symptomatic bradycardia, while amitriptyline at 50 mg/day was ineffective.

An indomethacin trial could not be adequately completed because of poor drug tolerance. Even at 25 mg three times daily with concomitant PPI therapy, the patient developed disabling nausea and dizziness, precluding further dose escalation. The trial was therefore considered negative. The final diagnosis was persistent headache attributed to mild traumatic injury to the head (ICHD-3 code 5.2.2), clinically mimicking probable hemicrania continua because of the absence of indomethacin responsiveness. Topiramate (100 mg/day) was well tolerated and resulted in an approximately 50% reduction in headache severity after six months, with occasional headache-free days.

**Patients 3 and 4**

Patients 3 and 4 were referred for refractory chronic migraine, both presenting with strictly left-sided headaches. Patient 3 had progressively developed a continuous unilateral background headache suggestive of hemicrania continua, whereas Patient 4 experienced ipsilateral lacrimation and rhinorrhea during migraine attacks, raising the possibility of paroxysmal hemicrania.

Because of the strictly unilateral phenotype, both patients underwent an indomethacin trial (150 mg/day with concomitant PPI therapy for one month). Neither patient experienced any clinical benefit; indeed, both reported a better response to ibuprofen and naproxen than to indomethacin. The final diagnosis was chronic migraine with central sensitization in Patient 3 and chronic migraine with prominent cranial autonomic symptoms in Patient 4. Both patients subsequently experienced clinically meaningful improvement with anti-CGRP monoclonal antibodies (Eptinezumab) combined with onabotulinumtoxinA administered according to the PREEMPT protocol.

**Patient 5**

A 34-year-old man with active tobacco and cannabis use, and occasional cocaine consumption, had a five-year history of cluster headache. Attacks consisted of strictly right-sided periorbital crushing pain lasting approximately 30 minutes, accompanied by ipsilateral eyelid oedema and marked restlessness. Headache bouts followed a circannual pattern, recurring predominantly during autumn with remission throughout spring and summer. During active bouts, attack frequency reached five episodes per day.

According to the patient, both subcutaneous sumatriptan and oxygen therapy acted too slowly to provide meaningful relief, while verapamil had been ineffective at 720 mg/day.

Because of the relatively short attack duration, paroxysmal hemicrania was considered in the differential diagnosis. An indomethacin trial with gradual dose escalation to 300 mg/day, combined with PPI therapy for one month, resulted in only a modest 20% reduction in attack frequency (from five to four attacks per day). The trial was therefore considered negative. Seasonal lithium prophylaxis combined with a right greater occipital nerve block subsequently achieved an approximately 50% clinical improvement. Final diagnosis was episodic cluster headache.

**Patient 6**

A 31-year-old woman with relapsing-remitting multiple sclerosis receiving natalizumab presented with recurrent episodes of severe strictly right-sided headaches evolving over several months. She had previously experienced two similar self-limited episodes lasting several months each.

Attacks lasted only a few minutes and were characterized by stabbing, electric shock-like pain involving the right ophthalmic and maxillary trigeminal divisions, recurring several times per hour. No trigger zones or precipitating factors were identified. Attacks were accompanied by ipsilateral lacrimation and intermittent right-sided ptosis. Restlessness was present during some attacks. A mild continuous background headache gradually developed during the course of the disease but had not been present initially.

An indomethacin trial (150 mg/day for one week followed by 225 mg/day for three weeks) failed to improve symptoms. The final diagnosis was secondary SUNA associated with multiple sclerosis. Lacosamide monotherapy (600 mg/day) resulted in an approximately 80% reduction in attack frequency and severity, although treatment was associated with daytime somnolence.

**Patient 7**

A 52-year-old man was referred for continuous pain involving the left mandibular division of the trigeminal nerve. The background pain was described as a constant pressure sensation interrupted by severe exacerbations lasting several hours and characterized as burning and tearing. No trigger zones or provoking factors were identified, and sensory examination was normal. Cranial autonomic symptoms were absent, although marked restlessness accompanied exacerbations.

Previous treatment with gabapentin, pregabalin, amitriptyline and duloxetine had been ineffective. Dental evaluation and dedicated MRI of the mandibular nerve revealed no abnormalities.

Because of the strictly unilateral pain, prolonged exacerbations and associated restlessness, an indomethacin trial was performed with dose escalation to 225 mg/day for four weeks. No clinical improvement was observed. The final diagnosis was painful idiopathic trigeminal neuropathy involving the left mandibular division. The patient subsequently achieved approximately 70% pain relief with combined lacosamide (300 mg/day) and topical lidocaine patches (700 mg), applied during daytime every third day.

**Supplementary Appendix – Clinical description of patients with positive indomethacin trials**

**Patient 1**

A 42-year-old woman with no relevant medical history or regular medication gradually developed a continuous strictly right-sided headache over a two-year period, confined to the right temporoparietal region. The background headache was described as a pressing pain of moderate intensity (3/10), resulting in insomnia and impaired concentration. Superimposed exacerbations occurred approximately once per month, lasting between one and four days. During exacerbations, pain became excruciating, described as crushing and electric shock-like. No cranial autonomic symptoms or restlessness were reported. The patient remained in a dark room because of severe photophobia and phonophobia, without nausea, vomiting or osmophobia.

Previous preventive treatments, including amitriptyline (75 mg/day), propranolol (160 mg/day) and candesartan (16 mg/day), were ineffective. Triptans failed to abort exacerbations, whereas ketoprofen and naproxen provided only transient partial relief, reducing pain intensity by approximately 50% for about two hours after administration. The patient was referred for consideration of onabotulinumtoxinA treatment because of suspected chronic migraine.

Given the strictly unilateral continuous headache, an indomethacin trial was initiated at 150 mg/day with concomitant proton pump inhibitor (PPI) prophylaxis. Complete resolution of the continuous background headache was achieved after eight days of treatment. Six months later, the patient remained well controlled on indomethacin 75 mg/day, with complete resolution of the background headache and only one 16-hour exacerbation occurring during a period of marked occupational stress, with migrainous features that do not exclude an associated comorbid migraine disorder and do not challenge the initial diagnosis.

**Patient 2**

A 79-year-old woman with a history of high-grade atrioventricular block requiring permanent pacemaker implantation and receiving multiple cardiovascular medications, including apixaban (5 mg twice daily), was referred for evaluation of persistent unilateral headache. She had also been diagnosed three years earlier with metastatic left apical lung carcinoma involving the right parietal and occipital lobes and had undergone immunotherapy together with stereotactic radiotherapy for brain metastases.

Approximately one year before presentation, she progressively developed a continuous right-sided headache involving the entire hemicranium. The background pain was described as pressing, with an intensity of 5/10, and was accompanied by daily exacerbations lasting several hours and reaching maximal intensity. Ibuprofen, paracetamol and tramadol were ineffective. Neither cranial autonomic symptoms nor restlessness were reported. Oral corticosteroids prescribed by the treating radiation oncologist failed to improve symptoms. A recent PET scan demonstrated no metabolic activity within the previously treated intracranial metastases.

Because of the patient's advanced age and anticoagulant treatment, an indomethacin trial was undertaken cautiously with PPI prophylaxis. Within one day of treatment initiation (25 mg three times daily), the patient reported at least a 50% reduction in headache severity. Following dose escalation to 50 mg three times daily one week later, complete headache remission was achieved.

Unfortunately, treatment had to be discontinued after one month because of symptomatic anaemia and dizziness, raising suspicion of gastrointestinal adverse events despite ongoing investigation (upper gastrointestinal endoscopy pending). Headache recurred the day after treatment withdrawal. Subsequent treatment with compounded melatonin (10 mg nightly) combined with topiramate (50 mg/day) achieved an approximately 50% reduction in symptoms. The final diagnosis was probable secondary hemicrania continua related to intracranial metastatic disease.

**Patients 3 and 4**

Patients 3 and 4 were 27- and 34-year-old men, respectively, both referred by community neurologists because of presumed treatment-refractory cluster headache.

Both patients had experienced stereotyped attacks for more than one year. Patient 3 presented with strictly left-sided attacks, whereas attacks alternated sides in Patient 4. Both described excruciating periorbital crushing pain lasting between 30 and 45 minutes. Patient 3 exhibited ipsilateral lacrimation, ptosis and conjunctival injection, whereas Patient 4 reported no cranial autonomic symptoms but consistently displayed marked restlessness during attacks. Attack frequency reached up to ten attacks per week in Patient 3 and one to two attacks per day in Patient 4.

Subcutaneous triptans were ineffective in both patients. Oxygen therapy provided only partial relief in Patient 4. Preventive treatment with high-dose verapamil (up to 720 mg/day) and lithium failed in both cases.

An indomethacin trial resulted in complete cessation of attacks in both patients. Complete response was achieved after four days at 150 mg/day in Patient 3 and after eleven days at 225 mg/day in Patient 4. Three months later, Patient 3 remained attack-free while receiving 75 mg/day of indomethacin. Patient 4 experienced immediate recurrence of attacks following dose reduction; however, symptoms remained well controlled after introducing compounded melatonin (10 mg nightly), allowing indomethacin to be reduced to 125 mg/day. Patient 3 reported symptom onset immediately following a major emotional stressor (relationship breakdown). Both patients were ultimately diagnosed with definite paroxysmal hemicrania.

**Patient 5**

A 43-year-old woman with no relevant medical history apart from untreated anxiety-depressive disorder initially experienced an episode of severe vertigo with a normal brain MRI. Several days later, she developed a continuous strictly right-sided headache involving the entire hemicranium. The background pain was described as a dragging sensation with an intensity of 4/10. Superimposed exacerbations reached maximal intensity, were crushing in quality, accompanied by recurrent vertigo, and lasted from several hours to several days. No restlessness was reported.

The patient denied cranial autonomic symptoms; however, neurological examination at the initial consultation revealed mild right-sided ptosis and ipsilateral conjunctival injection despite the absence of an ongoing exacerbation. Previous treatment with tramadol followed by oxycodone had been ineffective.

An indomethacin trial (150 mg/day with concomitant PPI prophylaxis) resulted in an approximately 90% clinical improvement after sixteen days, supporting the diagnosis of probable hemicrania continua (near-absolute response to indomethacin). Mild continuous right-sided discomfort persisted, together with rare exacerbations lasting a few hours, consistently triggered by alcohol consumption or psychological stress. Escalation to 225 mg/day was poorly tolerated because of marked fatigue and dizziness. Notably, the patient's vertigo resolved completely following initiation of indomethacin therapy.

**Patient 6**

A 36-year-old woman had been followed by a neurologist for persistent post-traumatic headache for four years after a moderate traumatic brain injury. Headache onset occurred four days after the injury. She experienced recurrent attacks without continuous background pain, occurring once daily or every other day and lasting from 30 minutes to several hours. Attacks were strictly unilateral, ipsilateral to the trauma, involving the right temporal region. Pain was severe, pulsatile, and associated with moderate photophobia and phonophobia together with marked nausea. No cranial autonomic symptoms were reported, although restlessness was occasionally observed during severe attacks.

Previous preventive treatments had been unsuccessful. Amitriptyline was discontinued because of poor tolerability, propranolol at the maximum tolerated dose was ineffective, and topiramate was avoided because of the patient's future pregnancy plans. During the preceding year, treatment with onabotulinumtoxinA had reduced attack frequency by approximately 30–40% for two months after each injection, followed by a clear wearing-off effect and no benefit during the third month of the injection cycle. Triptans and conventional NSAIDs provided only partial symptomatic relief.

An indomethacin trial, titrated to 225 mg/day, resulted in an approximately 80% reduction in attack frequency after 21 days of treatment, leading to revision of the diagnosis to probable secondary paroxysmal hemicrania attributed to moderate traumatic brain injury. Two months later, the dose had been successfully reduced to 150 mg/day while maintaining the same clinical efficacy. Discontinuation of onabotulinumtoxinA is planned, with subsequent reassessment of headache control.

**Patients 7 and 8**

Patients 7 and 8 were 33- and 52-year-old women, respectively, referred for longstanding unilateral facial pain previously diagnosed as idiopathic neuropathic pain after unsuccessful treatment with gabapentinoids, tricyclic antidepressants, and serotonin-noradrenaline reuptake inhibitors.

Both patients reported continuous background pain. Patient 7 described persistent right temporoparietal burning pain associated with tingling and painful cold sensations. Approximately every three to four months, prolonged exacerbations lasting around two weeks developed, during which pain intensified and spread to involve the entire right hemicranium. During exacerbations, ipsilateral facial erythema was documented photographically, without other cranial autonomic symptoms or restlessness.

Patient 8 described continuous left cheek and retroauricular pain characterized by tingling, burning and electric shock-like sensations. Superimposed attacks occurred several times daily, lasting only a few minutes, during which pain intensity increased without changes in location or quality. No cranial autonomic symptoms were reported, although the patient consistently described marked restlessness during attacks.

In Patient 7, indomethacin at 150 mg/day resulted in complete pain resolution within three days. Four months later, she remained pain-free while receiving 75 mg/day.

Patient 8 also achieved complete headache remission with indomethacin 150 mg/day, although only after one month of treatment. Dose escalation had previously been attempted but was limited by disabling dizziness. Multiple attempts to taper indomethacin, including adjunctive treatment with topiramate, compounded melatonin, gabapentin or pregabalin, consistently resulted in recurrence of symptoms within 24 to 48 hours, necessitating continuation of indomethacin therapy.

**Patient 9**

A 38-year-old man with severe atopic dermatitis in clinical remission presented with a nine-month history of strictly left-sided temporoperiorbital headache. The continuous background headache was pulsatile and of moderate intensity, with a marked circadian fluctuation, consistently worsening between 6:00 PM and midnight without a positional component.

Superimposed exacerbations reached an intensity of 7–8/10, lasting from a few minutes to several days and occurring unpredictably, with clusters of several attacks per day alternating with symptom-free periods lasting several weeks. During exacerbations—and occasionally outside of exacerbations—the patient exhibited ipsilateral lacrimation. Ipsilateral ptosis occurred less frequently, without conjunctival injection. Intermittent ipsilateral hemifacial sweating was also reported. No restlessness was observed. Moderate photophobia and phonophobia accompanied exacerbations, whereas nausea and vomiting were absent.

Brain MRI revealed a 1.7-cm ipsilateral temporopolar meningioma, considered an incidental finding. Given the strong clinical suspicion of hemicrania continua, an indomethacin trial was progressively escalated to 375 mg/day. A gradual but complete clinical response was achieved only after five weeks of treatment. The dose was subsequently reduced to 150 mg/day without recurrence of symptoms.

After approximately three months, treatment was discontinued because of persistent abdominal pain suggestive of gastric intolerance despite a normal upper gastrointestinal endoscopy. Headache recurred within 48 hours of indomethacin withdrawal but was considerably less severe than before treatment initiation. Subsequent treatment with topiramate (100 mg/day) combined with gabapentin (1,200 mg/day) achieved satisfactory symptom control. Melatonin was ineffective. At six-month follow-up, the patient reported only mild residual background pain (2/10) without further exacerbations.

**Patient 10**

A 35-year-old woman had been followed since the age of 20 years for typical chronic migraine without aura, characterized by side-shifting unilateral attacks. Her headaches had initially been well controlled with amitriptyline (25 mg/day) and propranolol (80 mg/day), while oral sumatriptan (100 mg) was consistently effective as acute treatment. Following her first pregnancy, at approximately 28 years of age, she experienced complete migraine remission, allowing gradual discontinuation of preventive therapy.

At the age of 33 years, four months after her third pregnancy, she developed a new headache phenotype consisting of prolonged attacks lasting several days. Pain became strictly left-sided, involving the entire left hemicranium, and was described as pulsatile with a pulling quality and maximal intensity. Attacks were accompanied by marked photophobia, phonophobia, nausea and vomiting. Neither cranial autonomic symptoms nor restlessness were reported.

Reintroduction of her previous preventive treatments, combined with eptinezumab and onabotulinumtoxinA administered according to the PREEMPT protocol, failed to provide meaningful improvement after one year of treatment.

Given the change in headache phenotype and its strict lateralization, an indomethacin trial was initiated with concomitant proton pump inhibitor prophylaxis. Treatment at 225 mg/day resulted in a marked clinical response after 23 days, with an approximately 80% reduction in attack frequency and a 50% reduction in the intensity of residual attacks leading to the diagnostic reclassification as probable paroxysmal hemicrania with prolonged attacks. At three-month follow-up, indomethacin had been successfully tapered to 150 mg/day, allowing discontinuation of all previous preventive treatments while maintaining satisfactory headache control.

**Patient 11**

A 43-year-old man with active tobacco use and chronic alcohol consumption (approximately two units per day) was referred for presumed occipital neuralgia of one year's duration.

He reported recurrent paroxysmal attacks of strictly right-sided occipital and upper cervical pain lasting between one and two hours. Pain was of moderate intensity and described as pulsatile or pressing rather than neuropathic in quality. Attacks occurred four to five times per week. During attacks, the patient was unable to remain still, suggesting marked restlessness, but denied cranial autonomic symptoms. Photophobia was present, whereas phonophobia, nausea and vomiting were absent. There was no apparent relationship between attacks and alcohol consumption or withdrawal.

Because of the attack duration, unilateral presentation and prominent restlessness, paroxysmal hemicrania was suspected. An indomethacin trial (150 mg/day) resulted in complete remission of attacks after nine days. The patient remained attack-free for five months while receiving the same dose, which was well tolerated.

Subsequently, however, he developed a gradual secondary loss of therapeutic response, with recurrence of attacks despite dose escalation to 375 mg/day. At the third follow-up visit, careful re-examination revealed ipsilateral symptoms had been misinterpreted by the patient, who in fact exhibited left-sided lacrimation and conjunctival injection contralateral to the pain, findings that he had not previously noticed.

Given the possibility of an atypical cluster headache that had initially responded to indomethacin, verapamil, lithium, subcutaneous sumatriptan and high-flow oxygen therapy were subsequently attempted without clinical benefit. Ipsilateral greater occipital nerve block with depot corticosteroids provided relief for only ten days. Partial improvement (approximately 60%) was eventually achieved with compounded melatonin combined with gabapentin (1,800 mg/day). Topiramate was discontinued because of mood-related adverse effects. The final diagnosis was definite paroxysmal hemicrania with secondary loss of response despite an adequate indomethacin trial.
